# Trends in *Clostridioides difficile* infection prevalence among pediatric cancer patients: A systematic review and meta-analysis

**DOI:** 10.1371/journal.pone.0333962

**Published:** 2025-10-15

**Authors:** Muluneh Assefa, Sirak Biset, Azanaw Amare, Wesam Taher Almagharbeh, Getu Girmay, Mitkie Tigabie

**Affiliations:** 1 Department of Medical Microbiology, School of Biomedical and Laboratory Sciences, College of Medicine and Health Sciences, University of Gondar, Gondar, Ethiopia; 2 Department of Medical and Surgical Nursing, Faculty of Nursing, University of Tabuk, Tabuk, Saudi Arabia; 3 Department of Immunology and Molecular Biology, School of Biomedical and Laboratory Sciences, College of Medicine and Health Sciences, University of Gondar, Gondar, Ethiopia; Debre Markos University, ETHIOPIA

## Abstract

**Introduction:**

Pediatric cancer patients are highly susceptible to *Clostridioides difficile* infection (CDI) due to immunosuppression, prolonged hospitalization, and antibiotic exposure. This study determined the global pooled prevalence of CDI among pediatric cancer patients.

**Methods:**

According to the Preferred Reporting Items for Systematic Reviews and Meta-Analyses (PRISMA) guidelines, 20 available articles published between 1985 and 2024 were included in this study. The extracted data from the relevant articles were analyzed using STATA version 17.0. The effect size estimate was computed using a random-effects model, considering a 95% confidence interval. The I^2^ statistic and Galbraith plot were used to confirm heterogeneity. Univariate meta-regression, sensitivity, and subgroup analyses were conducted to identify the source of heterogeneity. Egger’s test and a funnel plot were used to check for publication bias.

**Results:**

The pooled prevalence of CDI was 15.41% (95% CI: 10.57–20.24%), with high heterogeneity (I^2^ = 99.90%) and statistical significance (p < 0.001). The trend in the study year was a minimum prevalence of 0.96% in 2016–2020 (Brazil) and a maximum prevalence of 38.26% in 2007–2017 (USA). In the subgroup analysis, a relatively high prevalence of CDI was observed in Asia (23.23%; 95% CI: 17.44–29.01%) and prospective studies (20.64%; 95% CI: 14.25–27.03%), and studies included pediatric patients with hematologic, solid tumors, and hematopoietic stem cell transplantation recipients (18.34%; 95% CI: 8.05–28.63%). The test of group differences (p < 0.001) in the continent in subgroup analysis and sample size (p = 0.049) in univariate meta-regression were sources of heterogeneity between the effect sizes of the individual studies.

**Conclusion:**

There is a significant burden of CDI in pediatric cancer patients. These findings highlight the need for regular detection and targeted treatment of CDI, including drug-resistant strains, in cancer patients to minimize severe complications and mortality.

## Introduction

*Clostridioides difficile*, previously known as *Clostridium difficile*, is a major cause of healthcare-associated diarrhea, driven by the release of toxins A and B from toxigenic strains of the bacterium [1]. The clinical presentation of *Clostridioides difficile* infection (CDI) ranges from mild enterocolitis to severe cases involving toxic megacolon, sepsis, and death [2]. The incidence of CDI was highest in hospital-onset healthcare facility settings, with 5.31 cases/1000 admissions and 5.00 cases/10,000 patient-days [3]. Pediatric cancer patients are particularly vulnerable to healthcare-associated infections owing to factors such as prolonged hospital stay, excessive broad-spectrum antibiotic use, chemotherapy-induced mucosal damage, and immunosuppression [4]. Infection, especially with hypervirulent strains, poses a significant threat to pediatric cancer patients and hematopoietic stem cell transplantation (HSCT), potentially leading to increased length of hospital stay, treatment delays, and severe morbidity and mortality [5]. The number of new and recurrent CDI cases has increased over the past decade because of the presence of virulent strains [1]. Antibiotic exposure, chemotherapy, and prolonged hospitalization are the main risk factors for CDI development in pediatric cancer patients [6]. The burden of CDI in pediatric patients varies according to the type of cancer diagnosis and treatment [7].

Providing comprehensive data on the prevalence trends of toxigenic CDI in high-risk populations is crucial for developing effective prevention and management strategies. Therefore, this systematic review and meta-analysis aimed to determine the pooled prevalence of CDI among pediatric cancer patients from a global perspective.

## Methods

### Study design and reporting

The Preferred Reporting Items for Systematic Reviews and Meta-Analyses (PRISMA) guidelines were used to report the findings [8] (S1 File). The study protocol was registered in the International Prospective Register of Systematic Reviews (PROSPERO), with identification number CRD420251004334 and link https://www.crd.york.ac.uk/PROSPERO/view/CRD420251004334

### Literature search strategy

This study focused on the burden of CDI in pediatric cancer patients. The study used the COCOPOP (Condition (CDI), Context (global), and Population (pediatric cancer patients) paradigms to determine the suitability of the studies for meta-analysis. The search included all published studies, and the final search was conducted between March 25 and April 10, 2025. The following electronic databases were used: PubMed/Medline, Scopus, EMBASE, Google Scholar, Hinari, Web of Science, Science Direct, Cochrane Library, and African Journals Online to identify articles reporting the prevalence of CDI in pediatric patients with malignancies. The search terms alone or in combination with Boolean operators such as “OR” or “AND” were applied. The PubMed search strategy was as follows: ((((*Clostridioides difficile*) OR (*Clostridium difficile*)) OR (*C. difficile*)) AND ((((pediatric*) OR (pediatric*)) OR (child)) OR (children))) AND ((((((((oncology) OR (cancer)) OR (malignancy)) OR (neoplasm)) OR (leukemia)) OR (lymphoma)) OR (solid tumor)) OR (hematology)). A manual search of the literature and other reviews was conducted. The retrieved articles were imported into EndNote X9 bibliographic software manager (Clarivate Analytics, Philadelphia, PA).

### Study selection and eligibility criteria

Three authors (MA, MT, and AA) screened the titles and abstracts of the studies. The articles were then assessed for eligibility, and any disagreements between the authors were resolved through discussion. Although this study included published articles with no language, all available studies were presented in the English language. Studies with unclear results, case reports, communication, letters to editors, opinions, reviews, meta-analyses, or studies on populations other than cancer patients were excluded.

### Risk of bias (quality) assessment

Articles were retrieved for review, and relevant information was carefully extracted. The quality of the individual original studies was evaluated using the Newcastle-Ottawa scale. The evaluation tool comprises three main components. Five stars were awarded to the first section of the tool for methodological quality (sample size, response rate, sampling process, risk factors, and exposure determination). The tool also evaluates the comparability of studies with potential two-star scores. The outcomes and statistical tests were evaluated and awarded up to three stars. Finally, studies that achieved moderate (5–6 stars) to high (> 6 stars) quality scores were included in this systematic review and meta-analysis. Four authors (MA, MT, AA, and GG) assessed the quality of included studies (S2 File).

### Outcome of interest

The main outcome of the study was to determine the global trend in CDI prevalence among pediatric cancer patients.

### Data extraction

Data from individual studies were extracted by four authors (MA, MT, AA, and GG) using Microsoft Excel data extraction format (S3 File). The information collected from the eligible studies included authors, year of publication, study area, study design, age group, number of pediatric patients with oncological status, type of cancer, number of CDI cases, and *C. difficile* detection methods.

### Data analysis

Data were exported to STATA version 17.0 for statistical analysis. The pooled prevalence of CDI and 95% confidence intervals are visually displayed using a forest plot. Subgroup analysis was performed according to continent, study design, sample size, and type of malignancy. Heterogeneity between the included studies was evaluated using a Galbraith plot and an index of heterogeneity (I^2^ statistic) value of 0% = no heterogeneity, ≤ 25% = low, 25%−50% = moderate, 50–75% = substantial, and ≥ 75% = high [9]. In all pooled analyses, heterogeneity resulting from differences in effects across studies was determined using a random effects model. A sensitivity analysis of the effect of each study on overall prevalence was also conducted. Publication bias was statistically investigated using Egger’s test [10] and visual inspection of funnel plots. A p-value of less than 0.05 in Egger’s test was considered to indicate statistically significant publication bias. Univariate meta-regression analysis was performed to assess the effects of the sample size and publication year on CDI prevalence. The results are presented in the tables, text, and figures.

### Ethics statement

The study was conducted following PRISMA and PROSPERO guidelines. Since this study is a secondary review of original studies, ethics approval was not required.

## Results

### Search results

**Fig 1:**
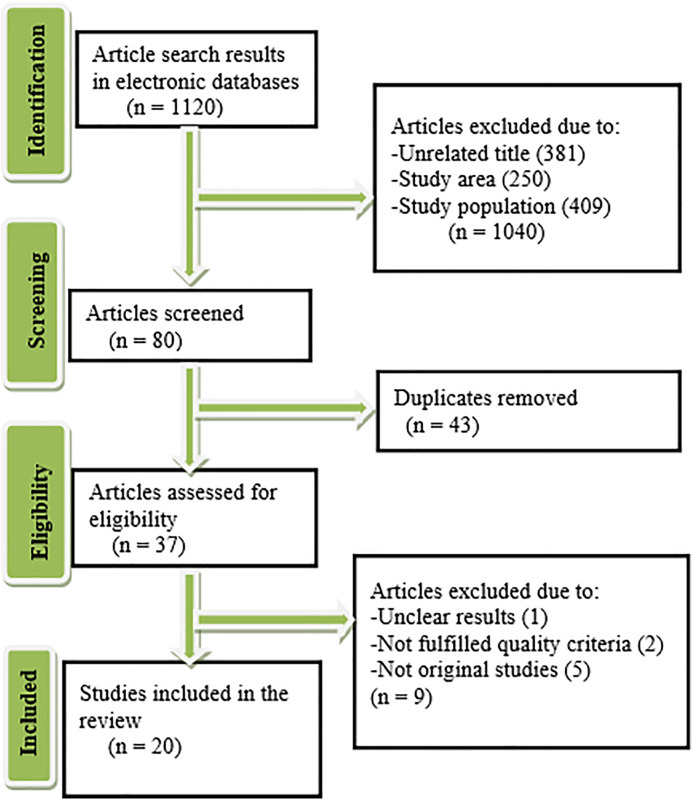
The flow diagram described the selection of studies.

In this study, 1,120 potentially relevant articles were identified. After reviewing the titles and abstracts, 43 articles were excluded because they were duplicates, and 37 articles were selected for further screening. Based on the evaluation of the exclusion/inclusion criteria and the quality of the articles, 20 articles were eligible for systematic review and meta-analysis [11–30] (Fig 1).

### Characteristics of the studies

Twenty studies comprising 62,547 pediatric cancer patients were included in this systematic review and meta-analysis. Twelve studies used retrospective study designs, while others used prospective designs. Eight studies were conducted in North America, seven in Europe, three in Asia, one in South America, and one in Africa (Table 1).

**Table 1:** Characteristics of studies reported on the prevalence of CDI among pediatric cancer patients.

Author, publication year	Study year	Study area	Study design	Age group	Patient population	*C. difficile* detection method	Sample size	*C. difficile* isolates	Prevalence of *C. difficile* infection (%)	Quality of studies using the Newcastle-Ottawa Scale
Lemiech-Mirowska et al, 2023	2020	Poland	Retrospective	5 months −15 years	Hematologic & solid tumor	Culture, Immunochromatographic assay	175	37	21.14	High
Silva et al, 2022	2016–2020	Brazil	Retrospective	0-18 years	Hematologic, solid tumor & HSCT	PCR, Toxin A/B Assay	6583	63	0.96	High
Al-Rawahi et al, 2018	2013–2016	Canada	Prospective	≤18 years	Hematologic, solid tumor & HSCT	PCR	276	92	33.33	High
Salamonowicz et al, 2018	2012–2015	Poland	Prospective	NA	Hematologic, solid tumor & HSCT	Culture, EIA, PCR	342	29	8.48	High
Dominguez et al, 2014	2012	USA	Prospective	11 months-21 years	Hematologic, solid tumor & HSCT	Culture, PCR (Toxin B gene)	45	10	22.22	Moderate
Tavafi et al, 2015	2011–2012	Iran	Prospective	≤15 years	Hematologic & solid tumor	PCR	105	18	17.15	High
Willis et al, 2020	2009–2018	USA	Retrospective	0.5 months – 24 years	Hematologic, solid tumor & HSCT	ELISA, PCR	952	109	11.45	High
Armin et al, 2013	2008–2009	Iran	Prospective	NA	Hematologic & solid tumor	Culture, ELISA(Toxins A&B)	152	38	25.00	High
Spruit et al, 2020	2007–2017	USA	Retrospective	NA	Hematologic, solid tumor & HSCT	PCR, Toxin A/B Assay	264	101	38.26	High
Simojoki et al, 2014	2007–2009	Finland	Retrospective	7 months-17 years	Hematologic, solid tumor & HSCT	Culture, EIA (Toxin A/B)	52	8	15.39	High
Castagnola et al, 2009	2005–2006	Italy	Retrospective	11 months-17.7 years	Solid tumor	Immunoenzymatic test	114	9	7.90	High
Daida et al, 2017	2003–2012	Japan	Retrospective	0-19 years	Hematologic & solid tumor	EIA (Toxin A)	189	51	26.98	High
Murphy et al, 2024	2000–2017	USA	Retrospective	1-18 years	Hematologic & solid tumor	PCR, EIA (Toxin A/B)	11366	206	1.81	High
Blank et al, 2013	1999–2011	USA	Retrospective	≤18 years	Hematologic & solid tumor	PCR, EIA	33095	1736	5.25	High
Fisher et al, 2014	1999–2009	USA	Retrospective	1-19 years	Hematologic	NA	8268	268	3.24	High
El-Mahallawy et al, 2001	1999–2000	Egypt	Prospective	1.5-18 years	Hematologic & solid tumor	EIA (Toxin A)	104	15	14.42	High
Price et al, 2013	1995–2004	Canada	Retrospective	≤18 years	Hematologic	NA	341	37	10.85	High
Oskarsdottir et al, 1991	1988–1989	Sweden	Prospective	NA	Hematologic & solid tumor	Culture	46	11	23.91	High
Brunetto et al, 1988	1986	UK	Retrospective	1-19 years	Hematologic & solid tumor	Culture	63	5	7.94	High
Chiesa et al, 1985	1982–1983	Italy	Prospective	4-14 years	Hematologic	Culture	15	4	26.67	Moderate

**Note. EIA; Enzyme Immuno Assay, ELISA; Enzyme Linked Immunosorbent Assay, PCR; Polymerase Chain Reaction, NA; Not Available.**

### Pooled prevalence of CDI among pediatric cancer patients

**Fig 2:**
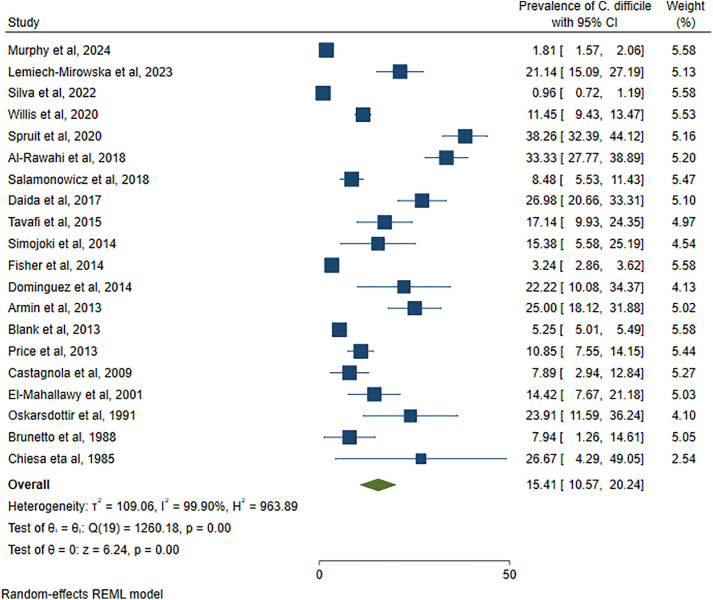
The forest plot showed the pooled prevalence of CDI among pediatric cancer patients.

**Fig 3:**
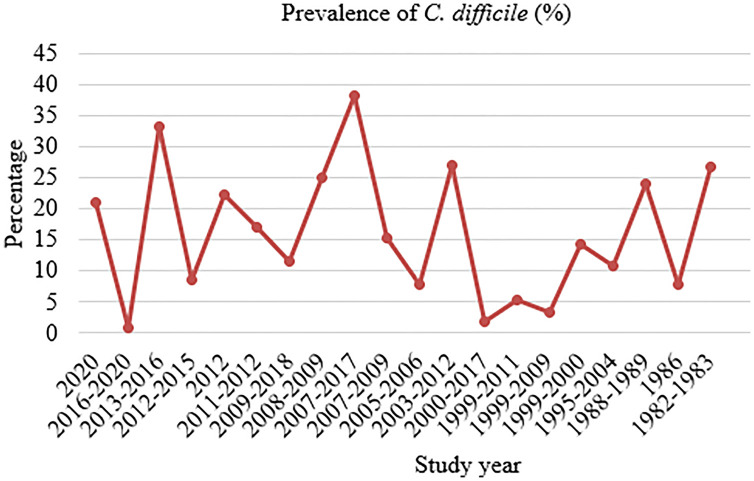
The figure shows the trends of CDI from 1982 to 2020.

Among 62,547 pediatric cancer patients, 2,847 had CDI. Accordingly, the pooled prevalence of *C. difficile* was 15.41% (95% CI: 10.57–20.24%), with high heterogeneity (I^2^ = 99.90%) and statistical significance (p < 0.001) (Fig 2). According to the study years of the individual articles, the minimum and maximum prevalence of CDI was reported to be 0.96% in 2016–2020 (Brazil) and 38.26% in 2007–2017 (USA) (Fig 3).

### Heterogeneity analysis

**Fig 4:**
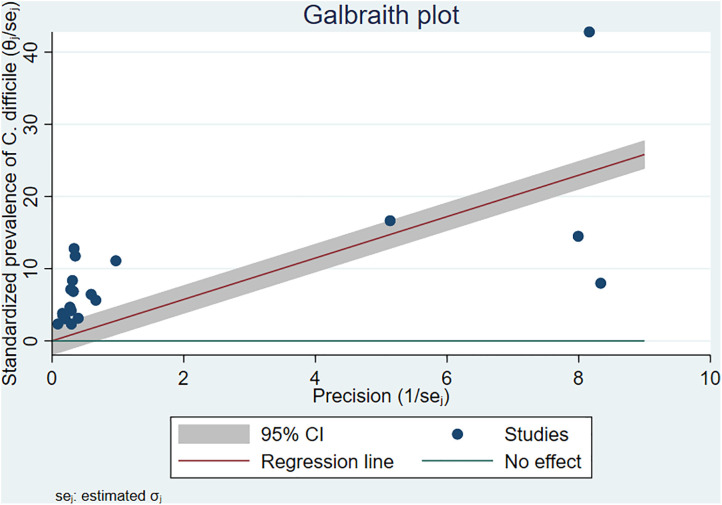
The Galbraith plot showed the heterogeneity between the studies.

According to the I^2^ result of 99.90%, as shown in the forest plot (Fig 2) and Galbraith plot (Fig 4), there was high heterogeneity among the included studies.

### Subgroup analysis

**Fig 5:**
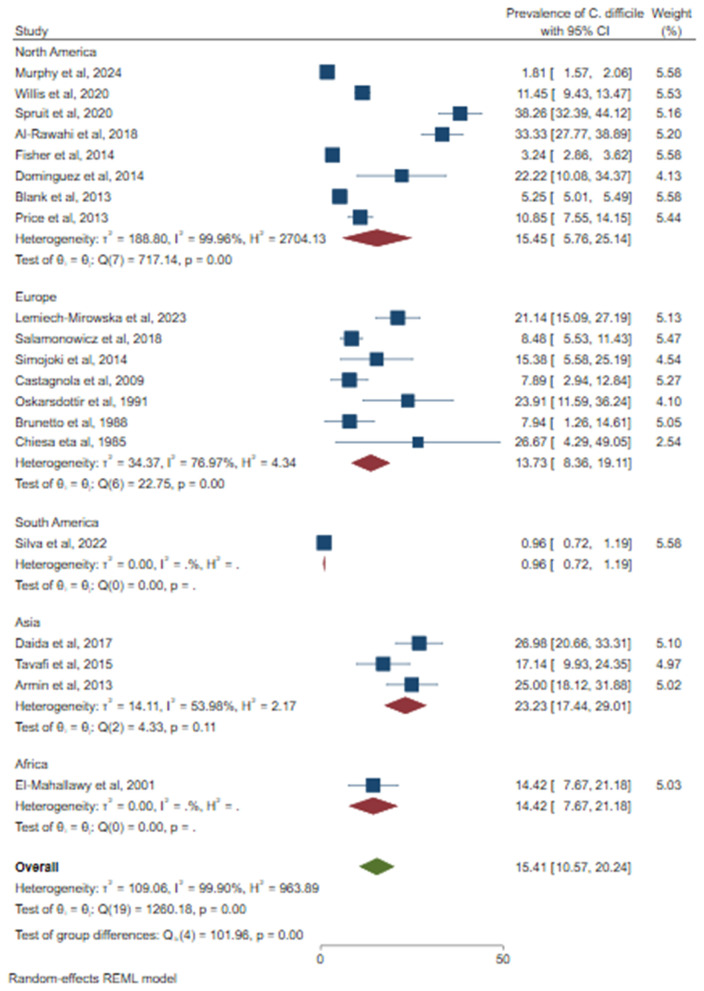
The forest plot showed subgroup analysis based on the continent.

**Fig 6:**
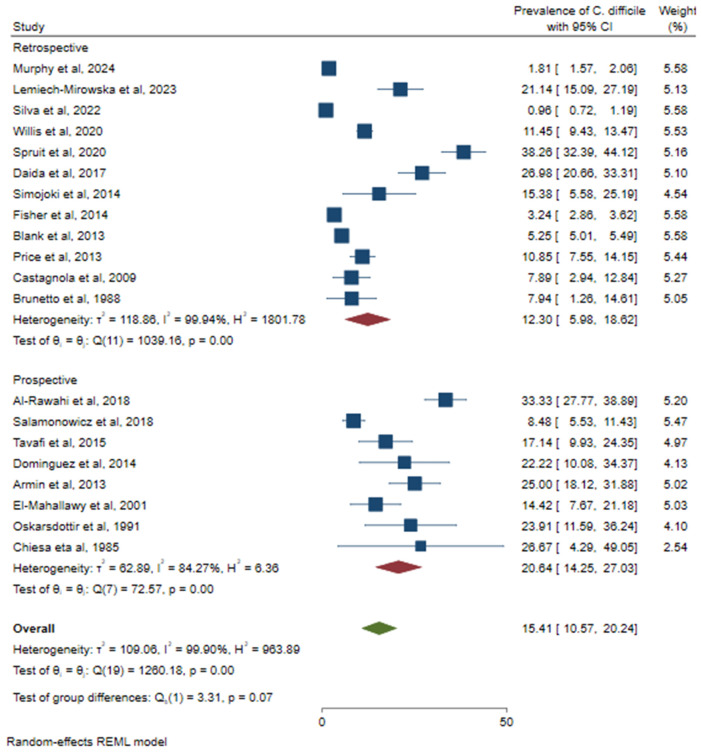
The forest plot showed subgroup analysis based on study design.

**Fig 7:**
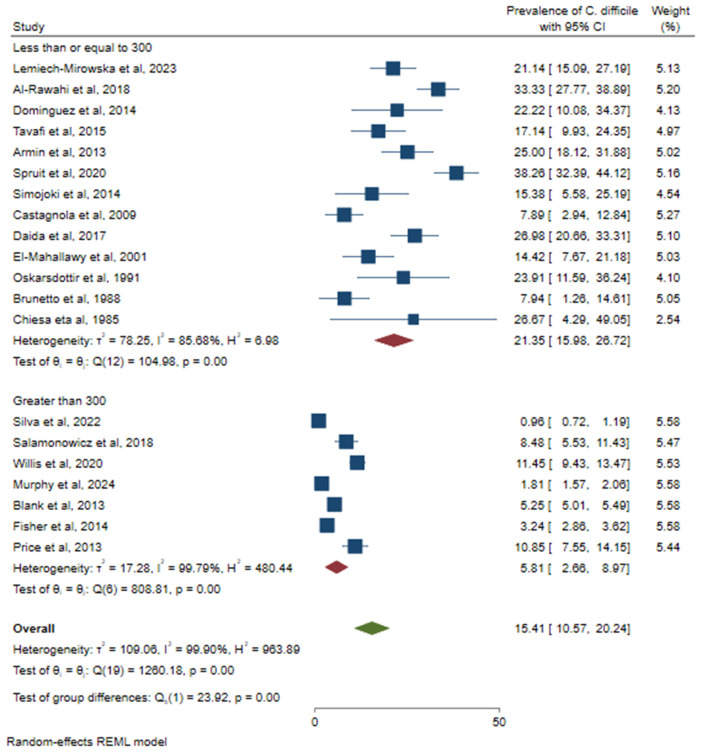
The forest plot showed subgroup analysis based on sample size.

**Fig 8:**
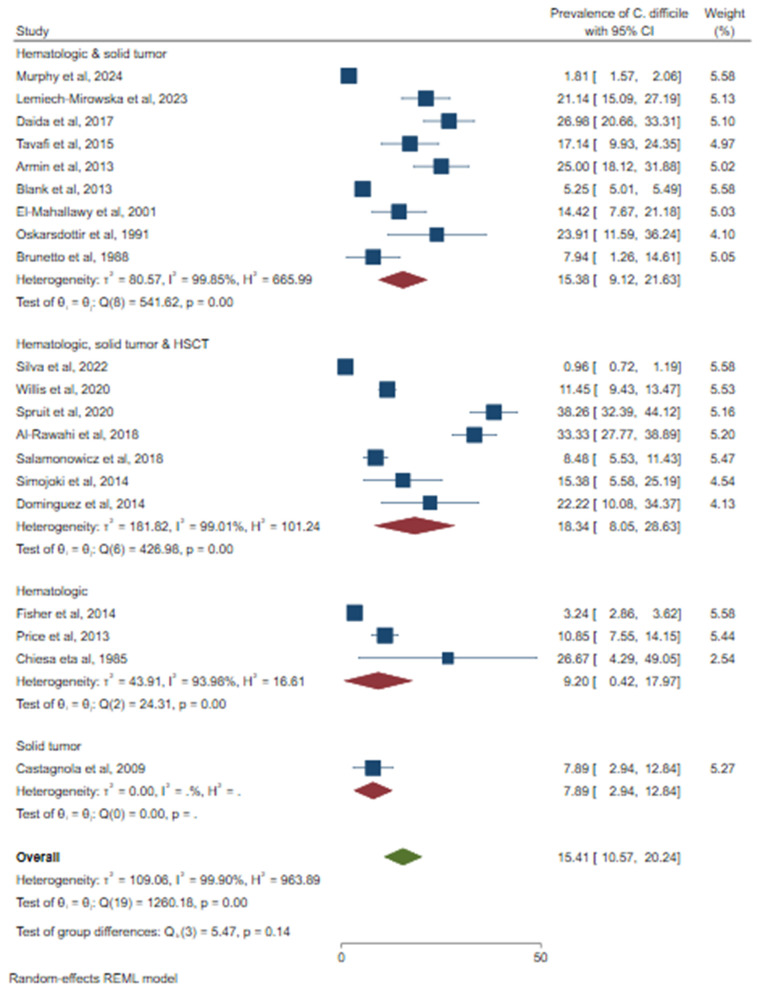
The forest plot showed subgroup analysis based on cancer type.

Because of the high heterogeneity of the studies, a subgroup analysis was conducted to identify the source of variation. A relatively high prevalence of CDI was observed in Asia (23.23%; 95% CI: 17.44–29.01%), followed by North America (15.45%; 95% CI: 5.76–25.14%) (Fig 5). Based on the study design, a higher prevalence of CDI was observed in prospective studies (20.64%; 95% CI: 14.25–27.03%) (Fig 6). Based on sample size, a relatively higher prevalence of *C. difficile* was observed among studies that used less than or equal to 300 (21.35%, 95% CI: 15.98–26.72%) (Fig 7). According to cancer type, the highest CDI was shown in studies including pediatric patients with hematologic and solid tumors and HSCT (18.34%; 95% CI: 8.05–28.63%), followed by both hematologic malignancies and solid tumors (15.38%; 95% CI: 9.12–21.63%) (Fig 8). The test of group differences revealed significant differences in effect sizes according to the continent (p < 0.001) and sample size (p < 0.001). However, the test of group differences in effect sizes was not statistically significant based on the study design (p = 0.07) and cancer type (p = 0.14).

### Publication bias

**Fig 9:**
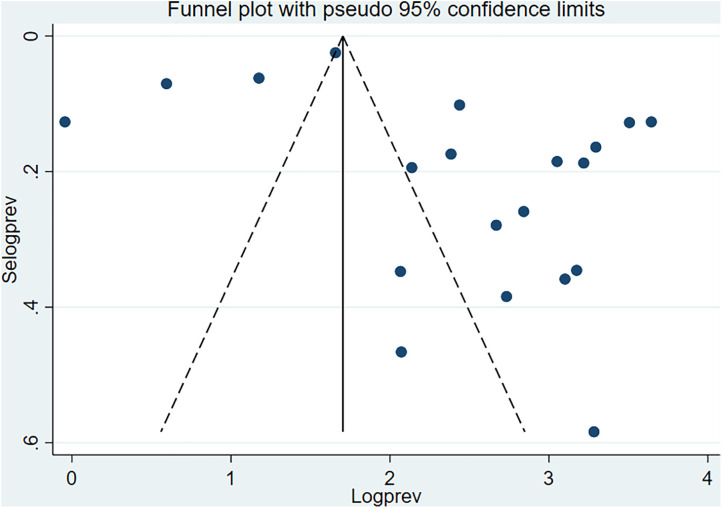
The funnel plot showed publication bias.

The presence of potential publication bias was statistically determined using Egger’s test. Egger’s test indicated significant publication bias (p = 0.008) (Table 2). Additionally, the graphical funnel plot showed an uneven distribution of the studies (Fig 9). While a formal test for publication bias was significant, the trim and fill analysis did not impute any studies. Therefore, the original pooled effect size is considered a robust estimate under this method. The asymmetry is due to true differences between studies of varying sizes and methodology, not a pattern of missing studies that cannot be corrected by the trim and fill analysis.

**Table 2:** Publication bias using Egger’s test.

Std_Eff.	Coef.	Std. Err.	t	p > t	[95% Conf. Interval]
Slope	2.05	0.53	3.85	0.001	0.93–3.16
Bias	5.34	1.80	2.97	0.008	1.57–9.11

### Sensitivity analysis

**Fig 10:**
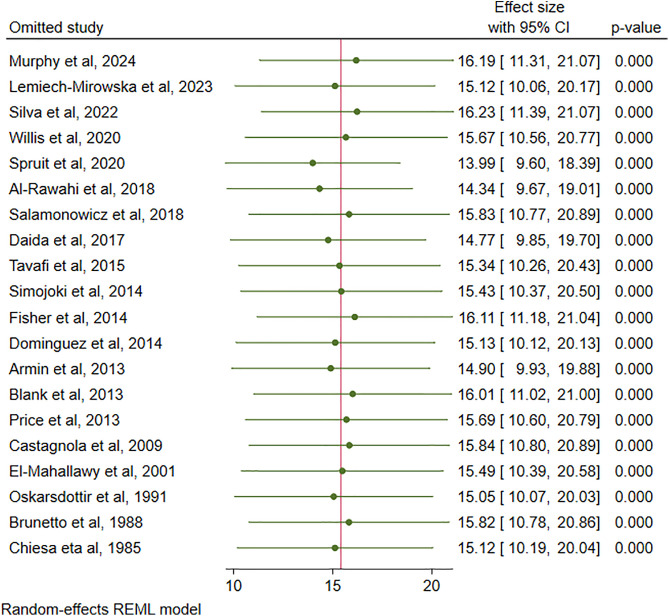
Sensitivity analysis.

Sensitivity analysis was performed to evaluate the extreme heterogeneity of the results. The step-by-step removal of each study was performed to determine the effect of each study on the pooled prevalence of CDI. The results showed that omitted studies had no significant effect on the pooled prevalence of CDI in pediatric cancer patients. The pooled estimate remains stable after removing low-quality studies (Dominguez et al and Chiesa et al), which indicates that the result is robust, reliable, and not driven by methodological weakness in a subset of the included research (**Fig 10).**

### Meta-regression

In this study, meta-regression analysis was conducted to determine the effect of sample size variation on the pooled prevalence of CDI. The results showed a significant association between the pooled estimate of CDI and the sample size (p = 0.049). Additionally, we determined the effect of publication year on CDI prevalence, and the results showed no statistically significant association (p = 0.841) (**Table 3**).

**Table 3:** Meta-regression by sample size and publication year.

Variables	Coef.	Std. Err.	t	p > t	[95% Conf. Interval]
Sample size	−0.0006	0.0003	−2.11	0.049	−0.001, −2.43
Publication year	−0.0503	0.2461	−0.20	0.841	−0.567, 0.47

## Discussion

The epidemiology of *C. difficile* has changed over the past 20 years, largely because of the emergence of hypervirulent and antimicrobial-resistant strains. The excessive use of antibiotics and poor infection control practices have resulted in the development of this significant health issue [31]. Oncologic patients are at an increased risk of CDI due to malignancy, cancer therapy, frequent antibiotic use, and a lower response rate to standard oral antibiotics [32]. This meta-analysis describes global trends in CDI among pediatric patients with cancer.

The overall pooled prevalence of CDI among pediatric patients with cancer was 15.41%. Similarly, various systematic reviews and meta-analyses have been conducted on the burden of CDI in different populations. Our finding is comparable with a meta-analysis reported 13.2% estimated prevalence of CDI among HSCT recipients, with 20.3% of CDI cases being severe [33]. A meta-analysis study on patients with COVID-19 reported CDI incidence rates ranged from 1.4 to 4.4 CDI cases per 10,000 patient days [34]. Another meta-analysis report on hospitalized patients with antibiotic-associated diarrhea showed a pooled estimated CDI prevalence of 20.0% [35]. According to the systematic review and meta-analysis in Ethiopia, the overall weighted pooled proportion of *C*. *difficile* among hospitalized diarrheal patients was 30.0% [36]. Moreover,7.8% of the hospitalized adult patients have asymptomatic colonization of *C. difficile* [37]. The overall prevalence of CDI in China was 11.4%. In line with the general situation in China, the most prevalent strains of *C. difficile* in southern China are ST54, ST3, and ST37. However, ST2 is the most common genotype in northern China [38]. On the other hand, a meta-analysis on the prevalence of community-acquired CDI stands at 5%, with an incidence rate of 7.3 cases per 100,000 person-years [39]. This indicates an increased burden of CDI in immunosuppressed individuals, such as pediatric oncology care settings, which requires screening practices, infection control, and antibiotic stewardship for cancer patients. The diagnostic method variability has a potential impact on the prevalence estimate of CDI. The conventional diagnostic methods, such as culture, could not provide more accurate prevalence results than the recommended advanced techniques, such as molecular detection of *C. difficile*.

Regarding heterogeneity, there was significant variation between the included studies (I^2 ^= 99.90%). Subgroup, meta-regression, and sensitivity analyses were performed to identify sources of heterogeneity. Although the number of studies varied, the results of the subgroup analysis revealed that the continent on which the study was conducted was significantly associated with effect size differences, with a relatively high prevalence of CDI in Asia, followed by North America, Africa, Europe, and South America. The frequency of CDI may be relatively high given the widespread uncontrolled use of antibiotics and incorrect prescriptions in many Asian countries. According to molecular studies, ribotypes 027 and 078, which have caused major epidemics worldwide, are rare in Asia. However, epidemics have been observed in variant toxin A-negative/toxin B-positive strains of ribotype 017 in different Asian countries [40,41]. According to the meta-regression analysis, differences in the sample sizes of individual studies had a significant effect on CDI prevalence. However, sensitivity analysis showed that the omitted studies did not have a significant effect on the pooled prevalence of CDI in pediatric cancer patients.

The trends of CDI among pediatric cancer patients varied according to the study year. The minimum prevalence was 0.96% between 2016–2020 (Brazil), whereas the maximum peak prevalence was 38.26% between 2007–2017 (USA), followed by 33.3% between 2013–2016 (Canada). A retrospective analysis of the US National Hospital Discharge Surveys from 2001–2010 among hospitalized adults reported nearly doubled CDI incidence, which increased from 4.5 CDI discharges per 1,000 total adult discharges in 2001 to 8.2 CDI discharges per 1,000 total adult discharges in 2010. In addition, mortality was increased slightly over the study period, from 6.6% in 2001 to 7.2% in 2010 [42]. The persistent increase in CDI exceeds other superbug pathogens in causing hospital-acquired infections. Recently, the Centers for Disease Control and Prevention mentioned CDI as an “urgent threat” in its current report on antibiotic resistance threats in the US, which requires urgent and special attention to prevent the infection [43]. The changes in the burden of CDI during recent years, with increases in incidence and severity of disease in several countries, have made CDI a global public health challenge. Increases in CDI prevalence have been mainly attributed to the emergence of highly virulent strains, increased toxin production, and high-level resistance to fluoroquinolones [44]. Surveillance systems are required to track trends and guide public health initiatives in light of these shifts in the epidemiology and microbiology of CDI. Since metronidazole is not an adequate treatment, faecal microbiota transplantation or the antibody bezlotoxumab are gaining importance in patients at risk or relapses [45]. Surveillance systems are required to track trends and guide public health initiatives in light of these shifts in the epidemiology and microbiology of CDI.

### Strengths and limitations of the study

This systematic review and meta-analysis is the first global report on CDI trends among pediatric cancer patients. However, information regarding the risk factors for CDI in pediatric oncology patients was not provided because of inconsistencies in the results reported by individual studies. The diagnostic method variability between the studies, such as culture, polymerase chain reaction, enzyme immunoassay, and enzyme-linked immunosorbent assay, may affect prevalence estimates. In addition, unclear data on the prevalence of CDI according to individual cancer type may be a potential source of bias. Moreover, due to the lack of published studies, not all continents of the globe were assessed, which would affect the generalizability of the CDI prevalence, but it indicated the gap for future research.

## Conclusion

This study reported a significant burden of CDI (15.41%) among pediatric cancer patients. In the subgroup analysis, a relatively high prevalence of CDI was observed in Asia, and the studies included pediatric populations with hematologic and solid tumors and HSCT. The recent increase in toxigenic and drug-resistant *C. difficile* isolates poses a risk to highly susceptible individuals, who require routine diagnosis and follow-up. Additionally, genomic characterization of drug-resistant and hypervirulent strains is crucial for the development of targeted treatments to minimize patient complications and mortality. Moreover, antimicrobial stewardship, infection control measures, and targeted surveillance in high-risk groups such as pediatric cancer patients are required.

## Supporting information

S1 File PRISMA checklist. (DOCX)

S2 File Quality assessment of the studies. (DOCX)

S3 File Data extraction format. (XLSX)

## Abbreviations

CDI: *Clostridioides difficile* infection
HSCT: Hematopoietic stem cell transplantation
PRISMA: Preferred Reporting Items for Systematic Reviews and Meta-Analyses
PROSPERO: International Prospective Register of Systematic Reviews
