# Supplementary material for: Trends in Clostridioides difficile infection prevalence among pediatric cancer patients: A systematic review and meta-analysis
Source: PLoS One. 2025 Oct 15;20(10):e0333962. doi: 10.1371/journal.pone.0333962 (PMC12527203; doi:10.1371/journal.pone.0333962)
Supplement: S2 File — (DOCX) [file pone.0333962.s002.docx]

**Supplementary file 2:** Methodological quality assessment of included studies using modified Newcastle - Ottawa Scale (NOS)

| Author | Criteria | | | | | | | |  |
| --- | --- | --- | --- | --- | --- | --- | --- | --- | --- |
|  | Selection | | | | Comparability | | Outcome | |  |
|  | Representativeness of the sample | sample size | Non –responders | Ascertainment of exposure/risk factor | The study controls for the most important factor | The study control for any additional factor | Assessment of the outcome | Statistical test | Quality score |
| Murphy et al, 2024 | A* | **A*** | **A*** | **B*** | A** | **-** | A* | **A*** | **8** |
| Lemiech-Mirowska et al, 2023 | A* | **A*** | **A*** | **B*** | **A*** | **-** | A* | **A*** | **7** |
| Silva et al, 2022 | A* | **A*** | **A*** | **B*** | A* | **-** | A* | **A*** | **7** |
| Willis et al, 2020 | A* | **A*** | **B*** | **A*** | A* | **B*** | A* | **A*** | **8** |
| Spruit et al, 2020 |  |  |  |  |  |  |  |  |  |
| Al-Rawahi et al, 2018 | A* | **A*** | **A*** | **B*** | A** | **-** | A* | **A*** | **8** |
| Salamonowicz et al, 2018 | A* | **A*** | **A*** | **B*** | A** | **-** | A* | **A*** | **8** |
| Daida et al, 2017 | B* | A* | B* | **A*** | **B*** | **--** | B* | A* | **7** |
| Tavafi et al, 2015 | A* | **A*** | **A*** | **B*** | A** | **-** | A* | **A*** | **8** |
| Simojoki et al, 2014 | A* | **A*** | **A*** | **B*** | A** | **-** | A* | **A*** | **8** |
| Fisher et al, 2014 | B* | A* | A* | **B*** | **B*** | **---** | --- | A* | **7** |
| Dominguez et al, 2014 | B* | B* | B* | **A*** | **B*** | **--** | B* | A* | **6** |
| Armin et al, 2013 | A* | **A*** | **A*** | **B*** | A** | **-** | A* | **A*** | **8** |
| Blank et al, 2013 | A* | **A*** | **A*** | **B*** | A** | **-** | A* | **A*** | **8** |
| Price et al, 2013 |  |  |  |  |  |  |  |  |  |
| Castagnola et al, 2009 | B* | A* | B* | **A*** | **B*** | **--** | B* | A* | **7** |
| El-Mahallawy et al, 2001 | B* | A* | B* | **A*** | **B*** | **--** | B* | A* | **7** |
| Oskarsdottir et al, 1991 | B* | A* | A* | **B*** | **B*** | **---** | --- | A* | **7** |
| Brunetto et al, 1988 | B* | A* | B* | **A*** | **B*** | **--** | B* | A* | **7** |
| Chiesa eta al, 1985 | B* | B* | **A*** | B* | B* |  | B* | A* | **6** |

Selection: (Maximum 5 stars)

1. Representativeness of the sample: A*) truly representative of the average in the target population (All subjects or random sampling)B*) somewhatrepresentative of the average in the target population (nonrandom sampling)C) Selected group of users D) No description of the sampling strategy
**2.** Sample size: A*) Justified and satisfactoryB) Not justified.
**3.** Non-respondents: A*) Comparability between respondents and non-respondents characteristics isestablished, and the response rate is satisfactoryB) The response rate is unsatisfactory, or the comparability between respondentsand non-respondents is unsatisfactory C) No description of the response rate or the characteristics of the responders andthe non-responders.

**4.** Ascertainment of the exposure (risk factor): A**) validated measurement tool. B) Non-validated measurement tool, but the tool is available or described C) No description of the measurement tool.
Comparability: (Maximum 2 stars)
1) The subjects in different outcome groups are comparable, based on the study designor analysis. Confounding factors are controlled. A*) The study controls for the most important factor (select one). B*) The study control for any additional factor.
Outcome: (Maximum 3 stars)
1) Assessment of the outcome: A**) Independent blind assessment B**,) Record linkage. C*) Self report D) No description.
2) Statistical test: A*) The statistical test used to analyze the data is clearly described andappropriate, and the measurement of the association is presented, includingconfidence intervals and the probability level (p value). B) The statistical test is not appropriate, not described or incomplete
